# Diagnosing Autism Spectrum Disorder: who will get a DSM-5 diagnosis?

**DOI:** 10.1111/jcpp.12085

**Published:** 2013-05-23

**Authors:** Rachel G Kent, Sarah J Carrington, Ann Le Couteur, Judith Gould, Lorna Wing, Jarymke Maljaars, Ilse Noens, Ina van Berckelaer-Onnes, Susan R Leekam

**Affiliations:** 1 Wales Autism Research Centre, School of Psychology, Cardiff UniversityCardiff, UK; 2 Institute of Health and Society, Newcastle UniversityNewcastle, UK; 3 Lorna Wing Centre, National Autistic SocietyBromley, UK; 4 University of Leuven–KU LeuvenLeuven, Belgium; 5 Leiden UniversityLeiden, The Netherlands

**Keywords:** DSM-5, diagnosis, ASD, DISCO

## Abstract

**Background:**

Introduction of proposed criteria for DSM-5 Autism Spectrum Disorder (ASD) has raised concerns that some individuals currently meeting diagnostic criteria for Pervasive Developmental Disorder (PDD; DSM-IV-TR/ICD-10) will not qualify for a diagnosis under the proposed changes. To date, reports of sensitivity and specificity of the new criteria have been inconsistent across studies. No study has yet considered how changes at the ‘sub domain’ level might affect overall sensitivity and specificity, and few have included individuals of different ages and ability levels.

**Methods:**

A set of DSM-5 ASD algorithms were developed using items from the Diagnostic Interview for Social and Communication Disorders (DISCO). The number of items required for each DSM-5 subdomain was defined either according to criteria specified by DSM-5 (Initial Algorithm), a statistical approach (Youden J Algorithm), or to minimise the number of false positives while maximising sensitivity (Modified Algorithm). The algorithms were designed, tested and compared in two independent samples (Sample 1, *N* = 82; Sample 2, *N* = 115), while sensitivity was assessed across age and ability levels in an additional dataset of individuals with an ICD-10 PDD diagnosis (Sample 3, *N* = 190).

**Results:**

Sensitivity was highest in the Initial Algorithm, which had the poorest specificity. Although Youden J had excellent specificity, sensitivity was significantly lower than in the Modified Algorithm, which had both good sensitivity and specificity. Relaxing the domain A rules improved sensitivity of the Youden J Algorithm, but it remained less sensitive than the Modified Algorithm. Moreover, this was the only algorithm with variable sensitivity across age. All versions of the algorithm performed well across ability level.

**Conclusions:**

This study demonstrates that good levels of both sensitivity and specificity can be achieved for a diagnostic algorithm adhering to the DSM-5 criteria that is suitable across age and ability level.

A diagnosis of autistic disorder (DSM-IV-TR, American Psychiatric Association., 2000) or childhood autism (ICD-10, World Health Organisation, 1993) is given when an individual has clinical impairments in social interaction, communication, and restricted and repetitive behaviours and interests. This diagnostic description is to be changed as part of the proposal for DSM-5 (Jan, 2011) and replaced by the new category of Autism Spectrum Disorder (ASD), represented by two dimensions of social communication and repetitive behaviours. However, questions have been raised about who will get a DSM-5 diagnosis; in particular, there have been concerns that the proposed DSM-5 criteria might exclude some individuals who currently receive a DSM-IV-TR or ICD-10 diagnosis (e.g. Wing, Gould, & Gillberg, 2011).

The main initial concern was that individuals might be excluded due to good specificity but poor sensitivity of the new DSM-5 criteria. For example, an epidemiological study of 8-year-old children by Mattila et al. (2011) reported a 54% reduction in diagnostic sensitivity relative to DSM-IV-TR if draft DSM-5 criteria (Jan, 2011) were strictly applied. Similarly, Gibbs, Aldridge, Chandler, Witzlsperger, and Smith (2012) reported a 23% reduction in sensitivity in 2- to 16-year-old children. Comparable reductions in sensitivity were reported for both low functioning adults (37%; Matson, Belva, Horovitz, Kozlowski, & Mamburg, 2012) and at-risk toddlers (47.9%; Matson, Kozlowski, Hattier, Hotovitz, & Sipes, 2012). It was further suggested that the new DSM-5 criteria might particularly exclude those with higher cognitive ability and those with atypical or Asperger-like presentations. For example, in a re-analysis of DSM-IV field trials with children and adults, the new DSM-5 criteria identified only 46% of individuals with Pervasive Developmental Disorder (PDD) and an IQ above 70, and sensitivity was low for those with Asperger Syndrome and Atypical autism (McPartland, Reichow, & Volkmar, 2012). Taheri and Perry (2012) also found that sensitivity varied by IQ; 89.7% of individuals with an IQ below 40, but only 22.2% of individuals with an IQ above 70 met criteria for DSM-5.

Not all studies using the proposed DSM-5 criteria have reported poor sensitivity. Two recent studies that mapped DSM-5 criteria using items from the Autism Diagnostic Interview-Revised (ADI-R; Le Couteur, Lord, & Rutter, 2003) and the Autism Diagnostic Observation Schedule (ADOS; Lord et al., 2000) reported good sensitivity (above .91). However, specificity was poor (0.53 for the ADI-R and 0.63 for ADI-R and ADOS; Heurta, Bishop, Duncan, Hus, & Lord, 2012) or not reported (Mazefsky, McPartland, Gastgeb, & Minshew, 2013).

Adjustments such as ‘relaxing’ the number of subdomains required to meet each domain to increase sensitivity, have also been made to the proposed DSM-5 criteria in several studies. According to these ‘relaxed’ criteria, individuals are required to meet two rather than three of the social-communication subdomains (e.g. Matson, Hattier, & Williams, 2012). Although this adjustment has been shown to improve sensitivity, particularly for higher functioning individuals, it can also have the effect of decreasing specificity relative to the original DSM-5 criteria (Frazier et al., 2012; Heurta et al., 2012; Mattila et al., 2011; Taheri & Perry, 2012).

Only one study to date reported good levels of both sensitivity and specificity without adjustment to DSM-5 rules (Frazier et al., 2012). In this study, items were selected from two parent-report questionnaires collected from the Interactive Autism Network, a large registry of siblings with at least one child diagnosed with ASD. The comparison group in this study consisted of siblings of the affected individuals, including those with and without conditions such as ADHD and anxiety disorder reported by parents. This sample, therefore, was not typical of a clinical comparison group and the reported specificity may be inflated compared with other studies.

In summary, the majority of studies investigating DSM-5 report either good specificity or sensitivity, with only one study to date reporting both within the same sample. One limitation of the existing literature is that data were typically collected according to DSM-IV-TR criteria and mapped to the DSM-5 descriptions (e.g. Taheri & Perry, 2012). These data, therefore, may not have included sufficient information to address the full range of behaviours described by DSM-5 (Swedo et al., 2012). Furthermore, the majority of existing literature has focused on children, and there is a clear need for work with adults (Heurta et al., 2012).

Ongoing prospective field trials for DSM-5 will help clarify many of the unresolved issues regarding who will qualify for a DSM-5 diagnosis. Meanwhile, the newly proposed DSM-5 diagnostic criteria for ASD can be investigated by exploring data not collected specifically for the purpose of diagnosis according to DSM-IV-TR/ICD-10. This study uses a diagnostic tool based on the concept of a spectrum of autistic disorders. This concept predated the earliest ICD and DSM criteria for autism (Wing, 1988; Wing & Gould, 1979), and is therefore not constrained by existing international diagnostic classifications. The Diagnostic Interview for Social and Communication Disorders (DISCO; Wing, Leekam, Libby, Gould, & Larcombe, 2002; Leekam, Libby, Wing, Gould, & Taylor, 2002) is a semistructured clinical interview that provides a profile of an individual's strengths, needs, and difficulties, including sensory symptoms and possible coexisting conditions such as motor coordination difficulties and ADHD. In addition, the DISCO enables algorithm diagnoses according to ICD and DSM. The original ICD-10 childhood autism algorithm was based on 88 DISCO-9 items and a set of rules specifying how these items convert into diagnostic outcome (Leekam et al., 2002). This algorithm, and its modified forms for DISCO-10 and DISCO-11, has good interrater reliability and discriminant validity (Leekam et al., 2002; Maljaars, Noens, Scholte, & van Berckelaer-Onnes, 2012; Nygren et al., 2009) and shows strong agreement with outputs from the ADI-R (Nygren et al., 2009) and ADOS (Maljaars et al., 2012).

The first aim of this study was to design and test the sensitivity and specificity of a new algorithm to assist in diagnosis according to the proposed DSM-5 ASD criteria using DISCO items. The second aim was to address the paucity of research with adults and specific concerns that the new criteria may lack sensitivity when diagnosing higher functioning individuals. To address this aim, detailed analysis was made of the proposed algorithm across ages (children, adolescents and adults) and ability levels.

To optimise the sensitivity and specificity of the proposed DSM-5 algorithm, attention was given not only to the two domains (A: Social-communication and B: Restricted, repetitive patterns of behaviour) but also to subdomains embedded within each of these domains (three in domain A and four in domain B; Appendix S1). In published studies, the number of items or behaviours included in each subdomain has varied, for example from three to 13 (Frazier et al., 2012). Authors have usually required only one or two items to meet criterion on each subdomain, as specified by different drafts of the DSM-5 criteria. However, this uniform approach may not always produce the best sensitivity and specificity either at the subdomain, domain nor algorithm level because the likelihood of an individual having one of 13 behaviours in a subdomain is greater than having one of just three.

When setting thresholds for subdomains, both sensitivity and specificity are important in order to maximise the clinical utility of the threshold to identify true ASD cases whilst minimising false positives. For the development of a new DISCO DSM-5 algorithm for ASD, several different algorithm versions were compared; each version of the algorithm included the same items, but the subdomain thresholds were set using three different criteria. The first applied the minimum requirements outlined in the proposed DSM-5 criteria as have been applied by previous studies (e.g. Heurta et al., 2012). Therefore, only one behavioural item was required per subdomain. The second applied a standardised statistic (Youden J) to identify the optimal threshold for both sensitivity and specificity. The Youden J statistic (Youden, 1950) has been used in previous research on diagnostic assessment of ASD (e.g. Cohen et al., 2010) and other areas of medicine (e.g. Chiu et al., 2011; Portalez et al., 2012). The third involved selection of the highest number of behaviours that maintained the maximum sensitivity of the subdomain.

The goal was to compare the balance of sensitivity and specificity across these algorithms using two participant samples. As the first (Initial) algorithm approach relies purely on the overall algorithm rules (combining of subdomains) to exclude false positives, it is predicted to have excellent sensitivity but lower specificity. In contrast, the second (Youden J) algorithm approach should more evenly balance sensitivity and specificity for each subdomain, but may restrict sensitivity of the whole algorithm by controlling for specificity at the subdomain level and also through the combination of subdomains. The subdomain thresholds for the third (Modified) algorithm are raised as high as possible while maintaining maximum sensitivity. We therefore predict that this approach will have improved sensitivity relative to the second approach, and improved specificity relative to the first. The effect of relaxing the proposed DSM-5 criteria to two rather than three A subdomains (see Heurta et al., 2012; Mattila et al., 2011) was also tested and the sensitivity of each algorithm version across age and IQ was tested using an additional participant sample.

## Method

### Participants

Three datasets reported in previous studies were used for developing and testing the DSM-5 algorithms. Full details of participants' clinical and demographic characteristics can be found in previous reports for Samples 1 (Leekam et al., 2002; Wing et al., 2002), 2 (Maljaars et al., 2012) and 3 (Leekam, Libby, Wing, Gould, & Gillberg, 2000; Leekam, Nieto, Libby, Wing, & Gould, 2007). For Samples 1 and 2, clinical diagnoses of DSM-IV-TR Autistic Disorder or ICD-10 Childhood Autism were made before recruitment by an independent clinician who did not use the DISCO. A few children in each sample had diagnoses of Atypical Autism, Asperger Syndrome and PDD not otherwise specified (PDD-NOS). These individuals were combined with children with Autistic Disorder or Childhood Autism to form a single group. For both Sample 1 and 2, the grouping of higher and lower cognitive ability at the time of recruitment (IQ above or below 70, respectively) was confirmed using nonverbal standardised tests. For Sample 1, the Leiter International Performance Scale (Leiter, 1979) and the Bayley Scale for Infant Development (Bayley, 1993) were used and for Sample 2, the Dutch Test for Non-Verbal Intelligence (Tellegen, Winkel, Wijnberg-Williams, & Laros, 1998) was used. Sample 1 was matched on both chronological age and nonverbal IQ, while Sample 2 was matched on nonverbal mental age. Both samples included control groups of children with intellectual disability (ID) and typical development (TD) as comparisons for the high- and low-functioning ASD groups, respectively. In Sample 1, an additional group of children with a language impairment (LI) was included for comparison with the high-functioning ASD group. The DISCO interview was subsequently conducted by interviewers blind to clinical diagnosis.

The sample used for algorithm design (Sample 1) comprised parents of 82 children from the United Kingdom interviewed using DISCO-9 (Wing et al., 2002). Thirty-six children (34–140 months; 32 male) had a clinical diagnosis of autism; 18 had higher ability and 18 lower ability. The lower ability comparison group comprised 17 individuals with ID (40–140 months; 10 male). The higher ability comparison groups comprised 14 individuals with LI (49–136 months; nine male) and 15 TD children (51–135 months; nine male).

The validation sample (Sample 2) comprised parents of 115 children from the Netherlands interviewed using DISCO-11 (Dutch translation; van Berckelaer-Onnes, Noens & Dijkxhoorn, 2008). Fifty-two children (34–137 months; 43 male) had a clinical diagnosis of autism (17 higher ability, 35 lower ability). The higher ability comparison group included 37 TD children (24–49 months; 15 male) and the lower ability comparison group included 26 children with ID (48–134 months; 16 male).

Performance of the algorithms was examined across age and ability level in a third sample (Sample 3) drawn from the sample of 200 individuals reported in Leekam et al. (2000, 2007). IQ measures were primarily based on age-appropriate Wechsler Intelligence Scales, with participants divided into high- and low-ability groups (above and below IQ of 70) as described in Leekam et al. (2007). The DISCO was conducted with parents/carers during the diagnostic process. The updated (DISCO-11) algorithms for ICD-10 were run on the Sample 3 participants; only participants who met DISCO ICD-10 diagnostic criteria for childhood or Atypical autism were included. The final sample comprised 112 children (<144 months; 68 higher ability), 33 adolescents (144–216 months; 19 higher ability), and 45 adults (>216 months; 33 higher ability).

Informed parent consent at the time of interview enabled data to be used for current and future research. Subsequent ethical approval for use of the data was obtained from the university's Research Ethics Committee.

### Measures

The majority of DISCO items can be rated for present symptoms (current) and symptoms across life span (ever). Consistent with previous research, ever codes were used to develop the DSM-5 algorithm. For Samples 1 and 3, two DISCO-9 items were updated according to corresponding DISCO-11 items (Wing, 2006), so that the data from all samples were equivalent (according to DISCO-11).

### Item selection

The full set of 320 DISCO items were scrutinised in a three-stage process:

1. All DISCO items mapping onto DSM-5 descriptions (DSM-5 2011) were assigned to DSM-5 subdomains (Appendix S1) by two researchers with experience of ASD (RGK and SJC).
2. One clinician (JG) and researcher (SRL) with extensive knowledge of ASD and the DISCO reviewed item selection and placement. This resulted in the inclusion of four additional items (three in A1 and one in A2), movement of one item from A1 to A3 and deletion of one item from B2. The placement of three verbal items was queried between B1 and B2.
3. The proposed assignment of all items was reviewed by three experienced DISCO interviewers (two psychiatrists and one psychologist) based in Japan, Canada and the Netherlands. None had been involved in the study's design or implementation. All independently agreed on the placement of all items, giving separate consideration to placement of repetitive verbal items in B1 or B2. All decided that these items should be placed in B1.

As with the design of the ICD-10 algorithms (Leekam et al., 2002), codes for each item were selected that best met the description in the diagnostic guidelines. The majority of items in the DISCO are rated on a three-point severity scale: ‘marked’ when a behaviour occurs daily, when no strategy is in action, or whenever the opportunity arises; ‘minor’ when behaviours are less frequent or severe; or ‘no problem’. In line with standard diagnostic coding (e.g. established DISCO and ADI-R algorithms), the majority of items were scored as present (1) only if there was a ‘marked’ (severe) impairment.

The resulting set of 85 DSM-5 items (Appendix S1) had excellent overall internal consistency (Cronbach's alpha = .95), and 80% had good/excellent interrater reliability (kappa ≥ .7; Wing et al., 2002) in Sample 1.

### Setting algorithm thresholds

Each version of the new DISCO algorithm was based on the proposed DSM-5 criteria (accessed Feb, 2012; Appendix S1). These specify that individuals must meet all three subdomains from domain A (social-communication) and two of the four subdomains from domain B (repetitive behaviours). The threshold or number of items that must be present for an individual to ‘score’ on each subdomain differed between the algorithms. Threshold setting was conducted separately for each algorithm version using data from Sample 1 exclusively:

Initial Algorithm: All thresholds were set to one item as proposed by DSM-5, and in line with previous literature (e.g. Mattila et al., 2011).

Youden J Algorithm: Receiver Operating Characteristic (ROC) curves (a plot of sensitivity against 1-specificity) were used to identify the optimal threshold for each subdomain. The point at which each ROC curve maximally deviated from the chance line was calculated using the Youden J statistic [maximum = (sensitivity + specificity) − 1]. The thresholds selected for each subdomain according to this method can be seen in Appendix S2.

Modified Algorithm: This algorithm also used sensitivity and specificity values calculated from ROC curves; the threshold was selected that maximised specificity while maintaining the highest level of sensitivity (Appendix S2).

As in McPartland et al. (2012), the original ICD-10 abnormal early development criteria were adopted for domain C (early childhood onset manifesting when social demands exceed capacities). For the DISCO ICD-10 algorithm, at least one of seven possible items must be present (Leekam et al., 2002).

The ‘relaxed’ DSM-5 criterion was achieved for each version of the algorithm by reducing the threshold of domain A from all three to two or more of the three social-communication subdomains.

### Testing the algorithm

The three different sets of thresholds were developed using Sample 1 data. Each version of the algorithm was then tested in Sample 1 and in an independent validation sample (Sample 2). ROC curves were used to compute the sensitivity and specificity of each version of the algorithm, while the area under the curve (AUC) was calculated to quantify overall discriminative power. In Sample 3, the sensitivity of each version of the algorithm was measured against ICD-10 algorithm output. In all samples, McNemar's test was used to compare the proportion of individuals identified as ASD using each version of the algorithm with each of the other versions (Bonferroni corrections for multiple comparisons; *p* < .01). Additional chi-square analyses were conducted on Sample 3 to compare the sensitivity of each version of the DISCO DSM-5 algorithm across age-groups and across high- and low-functioning groups. Analyses for the prevalence of DISCO items across age and IQ in this sample were computed using chi-square tests (Bonferroni corrected for multiple comparisons within each subdomain).

## Results

Sensitivity and specificity for each coordinate of the ROC curve are reported in Appendix S2 for each subdomain. The sensitivity and specificity for the algorithm using each set of thresholds are presented in Tables 1 and 2.

**Table 1:** Table showing the sensitivity and specificity of the three proposed algorithms according to current DSM-5 rules (3/3 social and communication; 2/4 repetitive behaviours)

	Sample 1			Sample 2		
	Initial Algorithm	Youden J Algorithm	Modified Algorithm	Initial Algorithm	Youden J Algorithm	Modified Algorithm
LFA	18/18 (100%)	14/18 (77.78%)	18/18 (100%)	34/35 (97.14%)	24/35 (68.57%)	30/35 (85.71%)
HFA	18/18 (100%)	13/18 (72.22%)	18/18 (100%)	15/17 (88.24%)	5/17 (29.41%)	14/17 (82.35%)
ID	10/17 (58.82%)	1/17 (5.88%)	5/17 (29.41%)	6/26 (23.08%)	0/26	3/26 (11.54%)
LI	5/14 (35.71%)	1/14 (7.14%)	3/14 (21.43%)			
TD	0/15	0/15	0/15	0/37	0/37	0/37
All controls						
AUC	.84	.85	.91	.92	.78	.90
SE	.05	.05	.03	.03	.05	.03
Lower	.75	.76	.85	.87	.69	.83
Upper	.93	.95	.98	.98	.87	.97
Sensitivity	1	.75	1	.94	.56	.85
Specificity	.67	.96	.83	.91	1	.95
Clinical controls						
AUC	.76	.84	.87	.86	.78	.87
SE	.06	.05	.05	.05	.05	.05
Lower	.64	.74	.77	.75	.68	.77
Upper	.88	.94	.97	.96	.87	.96
Sensitivity	1	.75	1	.94	.56	.85
Specificity	.52	.94	.74	.77	1	.89

LFA, low-functioning ASD group; HFA, high-functioning ASD group; ID, intellectual disability; LI, language impairment; TD, typical development, AUC, area under the curve.

**Table 2:** Table showing the sensitivity and specificity of the three proposed DISCO DSM-5 algorithms according to relaxed DSM-5 rules (2/3 social and communication; 2/4 repetitive behaviours)

	Sample 1			Sample 2		
Initial Algorithm	Youden J Algorithm	Modified Algorithm	Initial Algorithm	Youden J Algorithm	Modified Algorithm
LFA	18/18 (100%)	16/18 (88.89%)	18/18 (100%)	34/35 (97.14%)	27/35 (77.14%)	34/35 (97.14%)
HFA	18/18 (100%)	18/18 (100%)	18/18 (100%)	16/17 (94.12%)	10/17 (58.82%)	16/17 (94.12%)
ID	12/17 (70.59%)	4/17 (23.53%)	9/17 (52.94%)	13/26 (50%)	1/26 (3.85%)	8/26 (30.77%)
LI	9/14 (64.29%)	2/14 (14.29%)	4/14 (28.57%)			
TD	0/15	0/15	0/15	1/37 (2.70%)	0/37	0/37
All controls						
AUC	.77	.91	.86	.87	.85	.92
SE	.05	.04	.04	.04	.04	.03
Lower	.67	.84	.78	.80	.77	.86
Upper	.87	.98	.94	.94	.93	.98
Sensitivity	1	.94	1	.96	.71	.96
Specificity	.54	.87	.72	.78	.98	.87
Clinical controls						
AUC	.66	.88	.79	.73	.84	.83
SE	.07	.05	.06	.07	.05	.06
Lower	.53	.78	.67	.60	.75	.71
Upper	.80	.97	.91	.86	.93	.94
Sensitivity	1	.94	1	.96	.71	.96
Specificity	.32	.81	.58	.50	.96	.69

LFA, low-functioning ASD group; HFA, high-functioning ASD group; ID, intellectual disability; LI, language impairment; TD, typical development, AUC, area under the curve.

### Specificity

Specificity was tested only in Samples 1 and 2 as Sample 3 did not include a comparison group. The results reported here are for the comparison between the ASD and clinical comparison groups. Specificity was highest in the Youden J algorithm in both Samples 1 and 2 and lowest for the Initial Algorithm (Table 1). The difference between these two algorithms was significant in Sample 1(χ^2^_(1)_ = 12.02, *p* < .01) but did not survive correction for multiple comparisons in Sample 2 (χ^2^_(1)_ = 5.04, *p* < .05). The Modified Algorithm improved specificity relative to the Initial Algorithm in both samples, but this effect was only significant in Sample 1(χ^2^_(1)_ = 6.04, *p* < .01). Moreover, the specificity of the Modified Algorithm was not significantly lower than the Youden J Algorithm in both samples (*p* > .01 in both samples). Relaxing the criteria decreased specificity for all three algorithms, although this effect was not significant in either sample (*p* > .01).

### Sensitivity

In all three samples, sensitivity was highest for the Initial Algorithm and lowest for the Youden J Algorithm (Tables 1 and 3). This difference was significant in all three samples (Sample 1: χ^2^_(1)_ = 8.03, *p* < .01; Sample 2: χ^2^_(1)_ = 19.01, *p* < .01; Sample 3: χ^2^_(1)_ = 39.01, *p* < 0.01). The sensitivity of the Modified Algorithm was not significantly different from the Initial Algorithm (*p* > .01 in all three samples) but was significantly greater than the Youden J Algorithm (Sample 1: χ^2^_(1)_ = 8.03, *p* < .01; Sample 2: χ^2^_(1)_ = 14.02, *p* < .01; Sample 3: χ^2^_(1)_ = 37.01, *p* < .01). Relaxing the criteria increased sensitivity for the Youden J Algorithm in all three samples; this effect did not survive correction for multiple comparisons in Sample 1 (Sample 1: χ^2^_(1)_ = 6.04, *p* < .05; Sample 2: χ^2^_(1)_ = 7.03, *p* < .01; Sample 3: χ^2^_(1)_ = 27.01, *p* < .01). The sensitivity of the other two algorithms was not improved by relaxing the criteria (*p* > .01 in all three samples).

**Table 3:** Sensitivity of algorithms across age and ability (high and low) in Sample 3

Ability	Children			Adolescents			Adults			Total
	High (68)	Low (44)	Total (112)	High (19)	Low (14)	Total (33)	High (33)	Low (12)	Total (45)	
Initial Algorithm	96%	98%	96%	90%	93%	91%	91%	100%	93%	95%
Youden J Algorithm	69%	84%	75%	58%	71%	64%	76%	83%	78%	74%
Modified Algorithm	96%	96%	96%	90%	93%	91%	88%	100%	91%	94%
Relaxed criteria										
Initial Algorithm	100%	98%	99%	95%	100%	97%	97%	100%	98%	98%
Youden J Algorithm	93%	93%	93%	79%	71%	76%	85%	92%	87%	88%
Modified Algorithm	100%	96%	98%	95%	100%	97%	91%	100%	93%	97%

### Effect of age and ability level

Relaxing the DSM-5 criteria did not significantly improve the sensitivity of the Initial or Modified Algorithms in any of the samples and specificity was reduced in Samples 1 and 2. Therefore, these two relaxed algorithm versions were not tested across age and ability in Sample 3.

The only version of the algorithm in which sensitivity varied significantly across age-group was the ‘relaxed’ Youden J Algorithm (χ^2^_(2)_ = 7.46, *p* < .05). Post hoc pairwise analyses revealed that this effect was driven by significantly reduced sensitivity for adolescents compared with children (Table 3; χ^2^_(1)_ = 7.59, *p* < .001). There was no significant effect of ability level on the sensitivity of any of the four algorithms.

Further analysis was conducted to identify items that were more commonly found in older or more able individuals. The majority of items (72%) were comparable across age and ability level (Appendix S1). Indeed three items were highly frequent (>90%) in both ability groups and child/adult age groups: sharing interests and enjoyment; friendships; and awareness of others' feelings. One additional item (does not interact with peers) was highly frequent in both children and adults. Nineteen items were identified with significantly different frequencies in the high- and low-functioning groups (Appendix S3); nine were more prevalent in the high-functioning ASD group than the low-functioning (reciprocal communication, interrupting conversations, anger towards parents, long-winded and pedantic speech, maintenance of sameness in routines, repetitive themes, insistence on perfection, collecting facts on specific subjects and repetitive activities related to special skills). Similarly, six had significantly different frequencies for adults compared with all individuals below 18 years old, with a further five when adults and children below 12 years were compared. Of these 11 items, six were more prevalent in adults (anger towards parents, imaginative activities, long-winded and pedantic speech, tone of voice, repetitive themes and collecting facts on specific subjects).

## Discussion

This study is one of the first to develop and validate a new DSM-5 algorithm from a single standardised diagnostic tool. Different versions of a new DISCO DSM-5 algorithm were developed and tested in three samples of data. Results using Sample 1 and 2 showed that the Initial Algorithm had the highest level of sensitivity but lowest specificity, while the Youden J Algorithm had the highest level of specificity but lowest sensitivity. The Modified Algorithm, which aimed to maximise specificity whilst maintaining the highest level of sensitivity for each subdomain had comparable sensitivity to the Initial Algorithm and comparable specificity to the Youden J Algorithm in both samples. Overall, the AUC was best for the Modified Algorithm. These results replicate and extend the findings of Frazier et al. (2012) by demonstrating good levels of both sensitivity and specificity of the DSM-5 criteria. The study adds new evidence to the debate that some individuals who currently meet DSM-IV-TR criteria may be missed by DSM-5, and the inclusion of adults addresses a clear limitation of the existing literature (e.g. Heurta et al., 2012). All algorithm versions performed comparably in high- and low-functioning individuals. Moreover, their performance was comparable for children, adolescents and adults with the exception of the ‘relaxed’ Youden J Algorithm. These results suggest that according to the DISCO DSM-5 algorithms, and particularly the Modified Algorithm, individuals across a broad range of age and abilities will receive a DSM-5 diagnosis.

One explanation for this finding is the range of items included in the algorithm. Endorsement for the majority of items was consistent across age and ability. Indeed, a small set of items were observed in above 90% of cases in the high- and low-ability groups and in both children and adults. In addition, a small minority of algorithm items were more relevant for higher functioning and older individuals. These items were more common in domain B, suggesting that items relating to restricted and repetitive language (e.g. long-winded and pedantic speech), repetitive activities related to a special skill, or collecting facts in a specific subject might identify higher functioning individuals and adults with ASD. The combination of these global, as well as more specific, items may have contributed to the inclusivity of the DISCO DSM-5 algorithms.

Comparison of the three algorithm approaches offered a transparent comparison of different methods of applying DSM-5 criteria and the results clearly demonstrate the importance of algorithm design. The thresholds set for each subdomain had a significant impact on the performance of the algorithm as a whole. This level of subdomain analysis has had little previous attention in the DSM-5 literature. Furthermore, until now, the solution to improve sensitivity in studies including higher functioning individuals with ASD has been to propose alterations to the DSM-5 rules (mostly through relaxing the criteria for domain A from 3/3 subdomains to 2/3), an approach that has typically resulted in good sensitivity (e.g. Heurta et al., 2012). In this study, application of the ‘relaxed’ DSM-5 criteria also did significantly improve sensitivity in the case of the Youden J Algorithm. However, it did not significantly increase the sensitivity of the Initial or Modified Algorithms. The sensitivity of the Relaxed Youden J Algorithm was still lower than the Modified Algorithm while the specificity of these two algorithms was comparable. Given that the purpose of the international classification systems such as DSM-IV and DSM-5 is to guide clinicians when making diagnoses, and that descriptions in DSM-5 should represent the current conceptualisation of ASD, we argue therefore that relaxing of criteria is less optimal than the use of an algorithm (e.g. the Modified Algorithm proposed here) that matches directly the pattern of behaviours specified by these guidelines.

Compared with some recent papers (e.g. Frazier et al., 2012; Heurta et al., 2012), the sample size in this study is relatively modest. Although a range of age and ASD symptoms were included in Sample 3, this is a clear limitation of the study and further work with other cases of ASD – including the female profile – across the age-span is needed. Moreover, large-scale representative population studies will be essential to clarify the capacity of the DSM-5 algorithms to differentiate ASD from other developmental disorders. A clear test of the validity of the DISCO DSM-5 algorithms will be their success when used in larger samples of individuals referred for assessment through standard clinical care pathways. Meanwhile, this study demonstrates that good levels of both sensitivity and specificity can be achieved for a diagnostic algorithm adhering to the DSM-5 criteria that is suitable across age and ability level.

**Key points**

- There are concerns that the proposed DSM-5 criteria will not identify the more able individuals currently diagnosed with high-functioning autism and Asperger syndrome.
- The reported sensitivity and specificity of the new criteria is inconsistent across studies and only one study has reported both good sensitivity and specificity in the same sample.
- The current research designed and compared different DSM-5 algorithm versions using items from the Diagnostic Interview for Social and Communication Disorders.
- Our results clearly demonstrate that algorithm design is an essential consideration when designing new diagnostic tools to meet the proposed DSM-5 criteria.
- Moreover, good sensitivity was reported across age and ability level, suggesting that the algorithms presented do not miss higher functioning individuals.

## Supporting information

**Appendix S1.** Draft DSM-5 criteria for Autism Spectrum Disorders (in bold) and DISCO algorithm items and subdomain thresholds in Sample 1. **Appendix S2.** ROC curve and subdomain thresholds. **Appendix S3.** Identifying the DISCO items that differ significantly between high- and low-ability individuals or between children and adults in Sample 3.
