# Supplementary material for: Diagnosing Autism Spectrum Disorder: who will get a DSM-5 diagnosis?
Source: J Child Psychol Psychiatry. 2013 May 23;54(11):1242–50. doi: 10.1111/jcpp.12085 (PMC4098079; doi:10.1111/jcpp.12085)
Supplement: Supplementary file 1 — Appendix S1. Draft DSM-5 criteria for Autism Spectrum Disorders (in bold) and DISCO algorithm items and subdomain thresholds in Sample 1. Appendix S2. ROC curve and subdomain thresholds. Appendix S3. Identifying the DISCO items that differ significantly between high- and low-ability individuals or between children and adults in Sample 3. [file jcpp0054-1242-sd1.docx]

***Online Appendix***

**Appendix S1**

**Table S1:** Draft DSM-5 criteria for Autism Spectrum Disorders (in bold) and DISCO algorithm items and sub-domain thresholds in Sample 1

|  | | **Frequency** | **Chi-square for domains and items** |
| --- | --- | --- | --- |
| **Criterion A - Persistent deficits in social communication and social interaction across contexts, not accounted for by general developmental delays, and manifest by ALL THREE of the following:** | |  | **52.77***** |
| **A1**  **Deficits in socio-emotional reciprocity, for example:** | |  | **52.77***** |
| *DSM-5 Example* | DISCO item |  |  |
| *from abnormal social approach and failure of normal back and forth conversation to response to total lack of initiation of social interaction* | Quality of social communication;  Makes one-sided social approaches*#  Seeks comfort when in pain or distress  Giving comfort to others  Inappropriate response to others’ emotions*  Avoidance of age peers# | 55.6  61.1  72.2  77.8  33.3  77.8 | 7.37**  34.80***  25.02***  34.87***  14.70**  23.49*** |
| *reduced sharing of interests, emotions, and affect* | Sharing interests and enjoyment*#  Reaction to others’ happiness  Emotionally expressive gestures#  Emotional response to age peers# | 88.9  38.9  50  77.8 | 24.36***  10.75***  20.04***  40.50*** |

| *Table S1 continued* | |  |  |
| --- | --- | --- | --- |
|  | | **Frequency** | **Chi-square for domains and items** |
| **A2**  **Deficits in non-verbal communicative behaviours used for social interaction, for example:** | |  | **25.02***** |
| *DSM-5 example* | DISCO item |  |  |
| *poorly integrated verbal and nonverbal communication* | Use of body language*#  Using other people as mechanical aids | 63.9  47.2 | 16.67***  9.99** |
| *abnormalities in eye contact and body-language* | Eye contact#  Brief glances#  Blank, unfocussed gaze#*  Stares too long and hard | 52.8  25  50  16.7 | 13.16***  7.42**  8.47**  1.20 |
| *deficits in understanding and use of nonverbal communication to total lack of facial expression or gestures* | Nonverbal communication*#  Understanding of gesture and miming  Facial expression  Instrumental gestures  Declarative gestures  (joint referencing)  Imperative gestures#  Descriptive gestures  Use of nodding and shaking head | 5.6  22.2  44.4  69.4  50  13.9  75  41.7 | 0.66  2.96  7.14**  13.78***  20.04***  6.80**  17.66***  12.33*** |
| *Table S1 continued* | |  |  |
|  | | **Frequency** | **Chi-square for domains and items** |
| **A3**  **Deficits in developing and maintaining relationships appropriate to developmental level (beyond those with caregivers), for example:** | |  | **3.29** |
| *DSM-5 example* | DISCO item |  |  |
| *difficulties adjusting behaviour to suit different social contexts* | Psychological barriers  Interrupting conversations#  Personal modesty  Anger toward parents†  Behaviour in public places  Embarrassing remarks in public#  Approaching strangers*# | 55.6  33.3  47.2  0  52.8  11.1  22.2 | 14.88***  4.85*  7.13**  /  9.91**  5.37*  .09 |
| *difficulties in sharing imaginative play and in making friends* | Imaginative activities  Friendship with age peers*#  Quality of friendship*  Conventions of peer interaction# | 44.4  97.2  13.9  27.8 | 11.95***  23.25***  1.29  3.86* |
| *apparent absence of interest in people* | Does not interact spontaneously with peers#  Lack of awareness of others’ feelings*#  Response to visitors* | 83.3  88.9  69.4 | 19.33***  29.98***  18.79*** |

|  | | **Frequency** | **Chi-square for domains and items** |
| --- | --- | --- | --- |
| **Criterion B – Restricted, repetitive patterns of behaviour, interests, or activities (manifest by at least TWO of the following):** | |  | **25.02***** |
| **B1**  **Stereotyped or repetitive speech, motor movements, or use of objects, for example:** | |  | **33.47***** |
| *DSM-5 example* | DISCO example |  |  |
| *simple motor stereotypies* | Rocking (standing up)  Complex movements  Unusual movements of hands or arms#  Self-spinning | 8.3  22.2  47.2  30.6 | 3.98*  11.33***  13.6***  2.77 |
| *echolalia* | Immediate echolalia  Delayed echolalia* | 44.4  61.1 | 5.90*  31.43*** |

| *Table S1 continued* |  |  |  |
| --- | --- | --- | --- |
|  |  | **Frequency** | **Chi-square for domains and items** |
| *repetitive use of objects* | Interest in parts of objects#  Elaborate repetitive activities with objects*  Abstract properties of objects*#  Quality of pattern activities | 16.7  25  27.8  58.3 | 3.48  2.85  8.87**  21.01*** |
| *idiosyncratic phrases and stereotyped speech* | Long winded pedantic speech  Tone of voice in speech *  Idiosyncratic use of words, signs*# | 8.3  33.3  13.9 | 3.98*  9.71**  4.09* |

| *Table S1 continued* | |  |  |
| --- | --- | --- | --- |
|  | | **Frequency** | **Chi-square for domains and items** |
| **B2**  **Excessive adherence to routines, ritualised patterns of verbal or on-verbal behavior, or excessive resistance to change, for example:** | |  | **16.09***** |
| *DSM-5 example* | DISCO item |  |  |
| *motoric rituals, insistence on same route or food* | Maintenance of sameness in routines#  Clinging to home or familiar places  Eats only a small range of foods*  Other repetitive routines#  Arranging objects# | 52.8  11.1  27.8  2.8  61.1 | 15.05***  2.82  5.19*  0.31  25.62*** |
| repetitive questioning | Repetitive questions  Repetitive themes  Repetitive acting out of roles | 36.1  25  13.9 | 3.72  5.52*  6.80** |
| extreme distress at small changes | Maintenance of sameness of environment*  Insistence on perfection | 44.4  30.6 | 14.00***  3.77 |

| *Table S1 continued* | |  |  |
| --- | --- | --- | --- |
|  | | **Frequency** | **Chi-square for domains and items** |
| **B3**  **Highly restricted fixated interests that are abnormal in intensity or focus, for example:** | |  | **10.05**** |
| *DSM-5 example* | DISCO item |  |  |
| *strong attachment to or preoccupation with unusual objects* | Fascinated with specific objects  Collecting objects  Clinging to objects | 69.4  25  30.6 | 13.78***  2.85  0.82 |
| *excessively circumscribed or perseverative interests* | Fascination with TV/videos*  Collecting facts on specific subjects  Activities related to a special skill | 66.7  11.1  30.6 | 15.1***  2.82  13.02*** |
| **B4**  **Hyper or hypo-sensitivity to sensory input or unusual interest in sensory aspects of the environment, for example:** | |  | **16.52***** |
| *DSM-5 example* | DISCO item |  |  |
| *apparent indifference to pain/heat/cold* | Indifference to pain heat or cold# | 27.8 | 8.87** |
| *adverse response to specific sounds or textures* | Distress caused by sounds* | 61.1 | 8.91** |
| *excessive smelling or touching of objects* | Smelling objects or people*#  Touching objects#  Repetitive, aimless manipulations of objects (not near eyes) as if seeking sensory stimulation | 22.2  38.9  30.6 | 11.33***  8.91**  10.40*** |
| *fascination with lights or spinning objects* | Fascination with sounds  Bright lights and shiny objects  Interest in watching things spin#  Twists hands or objects near eyes#  Interest in looking at objects from different angles | 11.1  19.4  19.4  22.2  33.3 | 1.36  1.19  4.71*  8.31**  14.70*** |

**(* *p*<.05, ** *p*<.01, *** *p*<=.001)**

* These items have very similar frequencies (within 5%) in both the high and low ability groups

# These items have very similar frequencies (within 5%) in both children and adults

† no individuals scored on “anger towards parents” in this Sample, although they do in Samples 2 and 3.

^The term Setback refers to behaviours in which the development of a skill initially follows a typical trajectory and then stops developing.

All items use the ‘ever’ codes where available except for items within Criterion C which do not have an ‘ever’ or ‘current’ code but instead an ‘age in months’ or ‘delay’ code.

**Appendix S2:** ROC curve and sub-domain thresholds


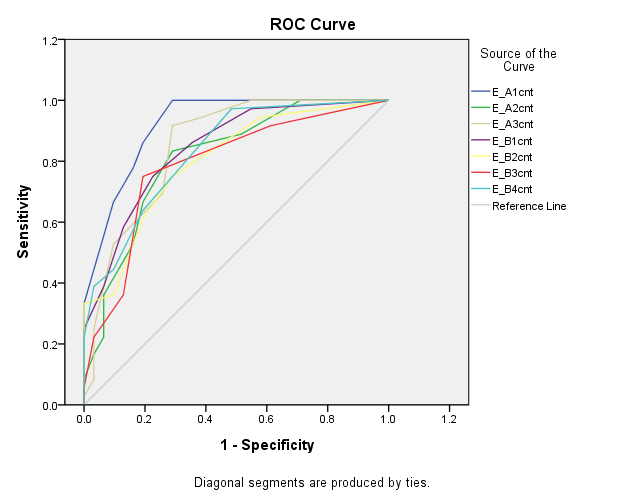


**Table S2:** Sub-domain thresholds

**A1**

| Threshold | Sensitivity | Specificity | Youden J |
| --- | --- | --- | --- |
| 1 | 1 | .258 | .258 |
| 2 | 1 | .484 | .484 |
| 3* # | 1 | .710 | .710 |
| 4 | .861 | .806 | .667 |
| 5 | .778 | .839 | .617 |
| 6 | .667 | .903 | .570 |

**A2**

| Threshold | Sensitivity | Specificity | Youden J |
| --- | --- | --- | --- |
| 1# | 1 | .290 | .290 |
| 2 | .944 | .387 | .331 |
| 3 | .889 | .484 | .373 |
| 4* | .833 | .710 | .543 |
| 5 | .667 | .806 | .473 |

**A3**

| Threshold | Sensitivity | Specificity | Youden J |
| --- | --- | --- | --- |
| 1 | 1 | .032 | .032 |
| 2 | 1 | .226 | .226 |
| 3# | 1 | .452 | .452 |
| 4 | .944 | .613 | .557 |
| 5* | .917 | .710 | .627 |
| 6 | .694 | .742 | .436 |

**B1**

| Threshold | Sensitivity | Specificity | Youden J |
| --- | --- | --- | --- |
| 1# | .972 | .452 | .424 |
| 2 | .861 | .645 | .506 |
| 3* | .750 | .774 | .524 |
| 4 | .583 | .871 | .454 |

**B2**

| Threshold | Sensitivity | Specificity | Youden J |
| --- | --- | --- | --- |
| 1# | .944 | .419 | .363 |
| 2* | .750 | .710 | .460 |
| 3 | .611 | .806 | .417 |
| 4 | .361 | .903 | .264 |

**B3**

| Threshold | Sensitivity | Specificity | Youden J |
| --- | --- | --- | --- |
| 1# | .917 | .387 | .304 |
| 2* | .750 | .806 | .556 |
| 3 | .361 | .871 | .232 |
| 4 | .222 | 968 | .190 |

**B4**

| Threshold | Sensitivity | Specificity | Youden J |
| --- | --- | --- | --- |
| 1*# | .972 | .516 | .488 |
| 2 | .639 | .806 | .445 |
| 3 | .444 | .903 | .347 |

* Threshold selected using the Youden J statistic; # Threshold selected for the modified DISCO algorithm

**Appendix S3**

| **Table S3:** Identifying the DISCO items that differ significantly between high and low ability individuals or between children and adults in Sample 3. | | | | | | | |
| --- | --- | --- | --- | --- | --- | --- | --- |
| Sub-domain | | DISCO item | % individuals scoring on DISCO item with significant chi-square | | | | |
|  | |  | IQ | | Age | | |
|  | |  | LFA | HFA | Children | Children & adolescents | Adults |
| A1 | Reciprocal Communication* | | 34.3 | 63.3 |  |  |  |
| A2 | Facial expression | | 75.7 | 54.2 |  |  |  |
| A2 | Descriptive gestures | | 82.9 | 62.5 |  |  |  |
| A2 | Nodding and shaking of head | | 58.6 | 30.8 |  |  |  |
| A2 | Understanding of gesture | | 35.7 | 7.5 | 25.9 | 22.8 | 2.2 |
| A2 | Using others as mechanical aids | | 54.3 | 27.5 | 49.7 |  | 20 |
| A3 | Psychological barriers | | 68.6 | 40.8 |  |  |  |
| A3 | Interrupting conversations* | | 40 | 61.7 |  |  |  |
| A3 | Personal modesty | | 75.7 | 40.8 |  |  |  |
| A3 | Anger toward parents Anger*# | | 4.3 | 25 | 11.6 | 12.4 | 33.3 |
| A3 | Imaginative activities# | | 68.6 | 44.2 | 43.8 |  | 71.1 |
| B1 | Rocking (standing up) | | 18.6 | 5 |  |  |  |
| B1 | Long winded pedantic speech*# | | 7.1 | 33.3 | 12.5 | 16.6 | 46.7 |
| B1 | Tone of voice # | |  |  | 41.1 |  | 66.7 |
| B1 | Self-spinning | |  |  | 35.7 |  | 11.1 |
| B2 | Maintenance of sameness in routines* | | 41.4 | 69.2 |  |  |  |
| B2 | Repetitive themes *# | | 27.1 | 53.3 | 29.5 | 37.9 | 62.2 |
| B2 | Insistence on perfection* | | 24.3 | 49.2 |  |  |  |
| B2 | Eats small range of foods | |  |  | 41.1 |  | 15.6 |
| B3 | Collecting facts on specific subjects *# | | 2.9 | 31.7 | 8 | 15.2 | 40 |
| B3 | Fascination with TV/videos | |  |  | 48.2 | 46.9 | 22.2 |
| B3 | Repetitive activities related to special skills* | | 14.3 | 31.7 |  |  |  |
| B4 | Twists hands or objects near eyes | | 24.3 | 8.3 |  |  |  |
| *Note.* *Items more frequent in high vs low ability # Items more frequent in adults than children | | | | | | | |
